# Pre‐Treatment Serum Albumin Concentration Predicts Clinical Benefit of Long‐Term Albumin in Patients With Cirrhosis and Ascites

**DOI:** 10.1002/ueg2.70258

**Published:** 2026-07-11

**Authors:** Enrico Pompili, Giacomo Zaccherini, Silvia Nardelli, Salvatore Piano, Carlo Alessandria, Sergio Neri, Francesco Giuseppe Foschi, Fabio Levantesi, Aldo Airoldi, Loredana Simone, Gianluca Svegliati‐Baroni, Stefano Fagiuoli, Fabio Marra, Raffaele Cozzolongo, Giulia Iannone, Maurizio Biselli, Oliviero Riggio, Paolo Angeli, Mauro Bernardi, Maurizio Baldassarre, Paolo Caraceni, for the ANSWER Study Investigators

**Affiliations:** ^1^ Department of Medical and Surgical Sciences Alma Mater Studiorum University of Bologna Bologna Italy; ^2^ Unit of Semeiotics, Liver and Alcohol‐related Diseases IRCCS Azienda Ospedaliero‐Universitaria di Bologna Bologna Italy; ^3^ Department of Translational and Precision Medicine Sapienza University of Rome Rome Italy; ^4^ Unit of Internal Medicine and Hepatology Department of Medicine—DIMED University and Hospital of Padova Padova Italy; ^5^ Division of Gastroenterology and Hepatology Città Della Salute e Della Scienza Hospital University of Turin Turin Italy; ^6^ Department of Clinical and Experimental Medicine University of Catania Catania Italy; ^7^ Internal Medicine Hospital of Faenza Azienda Unità Sanitaria Locale of Romagna Faenza Italy; ^8^ Internal Medicine Hospital of Bentivoglio AUSL of Bologna Bologna Italy; ^9^ Liver Unit Department of Hepatology and Gastroenterology Niguarda Hospital Milan Italy; ^10^ Gastroenterology Unit University Hospital Ferrara Italy; ^11^ Department of Gastroenterology Polytechnic University of Marche Ancona Italy; ^12^ Gastroenterology and Transplant Hepatology Papa Giovanni XXIII Hospital Bergamo Italy; ^13^ Department of Experimental and Clinical Medicine University of Florence Florence Italy; ^14^ Division of Gastroenterology National Institute of Gastroenterology S De Bellis Castellana Grotte (Bari) Italy

**Keywords:** decompensated cirrhosis, paracentesis, randomized controlled trial, refractory ascites, survival

## Abstract

**Background and Aims:**

The benefit of long‐term albumin (LTA) in improving survival and reducing complications in patients with cirrhosis and ascites is not consistently observed across studies, possibly reflecting differences in patient populations and treatment regimens. This study aimed to determine whether baseline serum albumin (SA) levels can predict which patients are most likely to benefit from LTA therapy.

**Methods:**

A post hoc analysis of the ANSWER trial was performed in 431 patients randomized to receive standard medical treatment (SMT) alone or SMT plus human albumin (SMT + HA). The interaction between baseline SA and LTA was investigated using competing‐risk survival analysis. The primary endpoint was 18‐month survival. Secondary endpoints included the incidence of cirrhosis‐related complications and hospitalizations.

**Results:**

A significant treatment‐by‐albumin non‐linear interaction was found (*p* = 0.010), indicating heterogeneity of treatment effect across baseline SA levels with the upper bound of the region of statistically demonstrable benefit occurring at approximately 3.2 g/dL (sHR 0.53, 95% CI 0.28–0.99). In patients with SA ≤ 3.2 g/dL, 18‐month survival was significantly higher in the SMT + HA group compared with SMT alone (HR 0.47, 95% CI 0.29–0.77; *p* = 0.0021). No significant survival difference could be demonstrated in patients with SA > 3.2 g/dL (HR 1.04, 95% CI 0.41–2.63; *p* = 0.93). Regardless of baseline SA levels, LTA was associated with improved ascites control and reduced rates of complications and hospitalizations.

**Conclusions:**

LTA provides survival and morbidity benefits in patients with mild‐to‐moderate hypoalbuminemia, whereas in patients with normal SA levels, its benefit appears mainly limited to morbidity reduction. Baseline SA may therefore help in prioritizing LTA therapy when resources are constrained.

## Key Summary

- Summarise the established knowledge on this subject
  ◦ Long‐term albumin administration has been shown to improve survival and reduce complications in some studies of decompensated cirrhosis with uncomplicated ascites, but results have been inconsistent across trials, possibly reflecting heterogeneity in patient populations and treatment regimens.
  ◦ The conflicting results have generated debate regarding the optimal use of this therapeutic strategy.
- What are the significant and/or new findings of this study?
  ◦ Long‐term albumin significantly improved 18‐month survival in patients with baseline mild‐to‐moderate hypoalbuminemia, whereas no survival advantage could be demonstrated in those with near‐normal levels.
  ◦ Conversely, albumin treatment significantly reduced the incidence of cirrhosis‐related complications and hospitalizations in all patients, regardless of their baseline albumin levels.
  ◦ Baseline serum albumin stratifies patients, identifying those achieving survival benefit versus morbidity control only. This may guide personalized treatment decisions and prioritize therapy allocation.

## 1 Introduction

Long‐term albumin administration (LTA) in patients with decompensated cirrhosis is a topic of interest and debate. In 2018, two large multicentre RCTs addressed this issue, reporting conflicting results. The ANSWER trial showed that the addition of weekly infusions of human albumin on top of diuretics (SMT) significantly improves overall survival and reduces the incidence of cirrhosis‐related complications in patients with uncomplicated ascites [1]. Conversely, the MACHT trial did not show such benefits [2]. Differences in study design, treatment duration, albumin dosing, and patient characteristics can explain these divergent findings [3]. More recently, an Italian multicentre real‐world observational study supported the efficacy of LTA, providing further evidence on clinical trajectories of patients, modalities and length of treatment, stopping criteria, and predictors of ascites resolution [4, 5]. Moreover, a few studies from Australia and India have supported the benefit of LTA in controlling ascites [6, 7].

However, LTA is still not widely used around the world in part because of cost and logistical challenges. Thus, identifying the subgroups of patients who are likely to derive the greatest benefit from this intervention could increase the cost‐effectiveness and consequently improve resource allocation. In this context, the use of easily available parameters that enable a more personalized treatment strategy remains an unmet need.

Serum albumin (SA) concentration has been constantly found to act as an independent predictor of poor clinical outcomes across many acute and chronic disease states, including cirrhosis [8]. In the ANSWER trial, the pre‐treatment SA concentration was closely correlated with the 18‐month survival in the control group, while this relationship was almost completely effaced in the group receiving LTA [1]. However, whether pre‐treatment SA concentration can be used to predict the clinical benefit from LTA treatment was not further investigated.

We addressed this open issue by conducting a secondary analysis of the ANSWER database, assessing the relationship between pre‐treatment SA concentration and mortality, ascites response, and incidence of complications of cirrhosis, with the objective of identifying the patients who had the greatest benefit from LTA.

## 2 Methods

### 2.1 Patients and Study Design

The present study is a post hoc analysis of the Human Albumin for the treatmeNt of aScites in patients With hEpatic ciRrhosis (ANSWER) trial—an investigator‐initiated, multicentre, randomized, parallel‐group, open‐label, pragmatic study conducted in 33 Italian academic and non‐academic hospitals [1]. The study protocol was approved by the ethics committee at the coordinating center and at each participating center. Written informed consent was obtained from all participants according to the 1975 Declaration of Helsinki. The study was registered with EudraCT (2008‐000625‐19) and ClinicalTrials.gov (NCT01288794). A detailed study protocol of the ANSWER trial has been previously published [1].

Eligible patients were adults with decompensated cirrhosis and persistent non‐complicated grade 2‐3 ascites despite ongoing diuretic treatment of at least 200 mg/day of an anti‐aldosteronic drug and 25 mg/day of furosemide. The main exclusion criteria were the presence of refractory ascites, recent acute complications of cirrhosis, previous Transjugular intrahepatic portosystemic shunt (TIPS) and ongoing alcohol abuse. The complete list of exclusion criteria is reported elsewhere [1].

A total of 431 patients were randomized to either standard medical treatment (SMT) (*n* = 213), or SMT combined with human albumin infusion (20% HA in 50 mL vials: 40 g twice weekly for two weeks and then 40 g weekly) (*n* = 218) up to a maximum of 18 months. The study was interrupted when patients underwent LT or TIPS insertion, needed three or more large volume paracenteses (LVP) per month, refused to continue their participation in the study, or because of medical judgment. Details on the study procedures have been published elsewhere [1].

### 2.2 Outcomes

The primary endpoint of the ANSWER trial was 18‐month overall survival. The following secondary endpoints were also assessed in the present post hoc analysis: incidence of paracentesis and refractory ascites and incidence of cirrhosis‐related complications including spontaneous bacterial peritonitis (SBP), other bacterial infections, renal impairment defined as the finding of serum creatinine concentration > 1.5 mg/dL, type‐1 hepatorenal syndrome (HRS) according to the 2003 definition of the International Club of Ascites [9], grade III/IV hepatic encephalopathy (HE), electrolyte disorders defined as episodes of hyponatremia (serum sodium concentration < 130 mmol/L) or hyperkalaemia (serum potassium concentration ≥ 5.5 mmol/L), and gastrointestinal bleeding related to portal hypertension.

Each endpoint was additionally compared between two patient subgroups defined by the exploratory baseline SA threshold derived from the interaction model to facilitate clinical interpretation.

### 2.3 Statistical Analysis

The normality of distributions and variance homogeneity were assessed for all continuous variables by the Shapiro‐Wilk and Levene tests. Variables were reported as mean and standard deviation (SD) or median and interquartile range (IQR) as appropriate. Comparisons between groups were performed using the Student's t‐test or the Mann‐Whitney *U* test as appropriate. Categorical parameters were reported as frequency and percentage and compared using the Chi‐square or Fisher exact test. Crude 18‐month mortality was summarized according to the tertiles of baseline serum albumin concentration and treatment arm.

The association between baseline serum albumin (SA) and mortality was assessed in the overall population using a multivariable Fine and Gray competing‐risk regression model, treating liver transplantation (LT) as a competing event for death. Baseline SA was modeled continuously using restricted cubic splines with three knots placed at the 10th, 50th, and 90th percentiles, and a treatment‐by‐baseline SA interaction term was included to account for potential non‐linear effect modification. The model was adjusted for key prognostic covariates, according to the framework of the ANSWER trial, including age, viral etiology of cirrhosis, MELD‐Na score, and Child–Pugh score [1]. Internal validation was performed using 1000 bootstrap samples.

The association between on‐treatment SA and mortality as well as its interaction with treatment was evaluated using the same competing‐risk approach in a 1‐month landmark analysis including patients still on follow‐up at day 30. In this analysis, 1‐month SA was modeled using restricted cubic splines with three knots placed at the 10th, 50th, and 90th percentiles, and a treatment‐by‐1‐month SA interaction term was included. The model was adjusted for MELD‐Na score assessed at the 1‐month visit [10].

Overall survival was estimated according to the Kaplan‐Meier method and compared by the Log‐Rank test. The incidence of complications was assessed by calculating the incidence rates (IRs) and then the incidence rate ratios (IRRs) using SMT as the reference group. Ninety‐five percent confidence intervals (CI) were computed using the exact method based on the Poisson distribution.

Fine and Gray competing‐risk regression models, treating LT as a competing event, were also used to estimate univariate subdistribution hazard ratios (sHRs) for 18‐month mortality for baseline anthropometric, clinical and laboratory parameters. All parameters significantly associated with 18‐month mortality with an alpha level of 5% were entered into a multivariable model with backward selection of predictors.

All tests were two‐sided and values of p less than 0.05 were considered statistically significant. Statistical analysis was performed using STATA (StataCorp LLC, College Station, TX, USA) version 18.

## 3 Results

### 3.1 Relationship Between SA Concentration and 18‐Month Mortality

Baseline characteristics of the 431 patients included in the ANSWER trial are reported elsewhere [1]. Notably, patients randomized in the SMT + HA or SMT arms did not differ regarding baseline SA concentration (3.1 ± 0.5 vs. 3.1 ± 0.6 g/dL, respectively; *p* = 0.857) [1].

When patients were stratified by tertiles of baseline SA concentration, crude 18‐month mortality showed the largest absolute difference between treatment arms in the lowest SA tertile, while differences were smaller in the intermediate and highest tertiles (Table 1).

**TABLE 1:** Crude 18‐month mortality according to tertiles of baseline serum albumin concentration and treatment arm.

	SMT + HA deaths, *n*/*N* (%)	SMT deaths, *n*/*N* (%)
1st tertile (SA ≤ 2.84 g/dL)	16/77 (20.8%)	21/68 (30.9%)
2nd tertile (SA > 2.84 to ≤ 3.30 g/dL)	14/74 (18.9%)	16/72 (22.2%)
3rd tertile (SA > 3.30 g/dL)	8/67 (11.9%)	9/73 (12.3%)

*Note:* Data are reported as number of deaths/total number of patients (%).

Abbreviations: HA: human albumin; SA: serum albumin; SMT: standard medical treatment.

**FIGURE 1:**
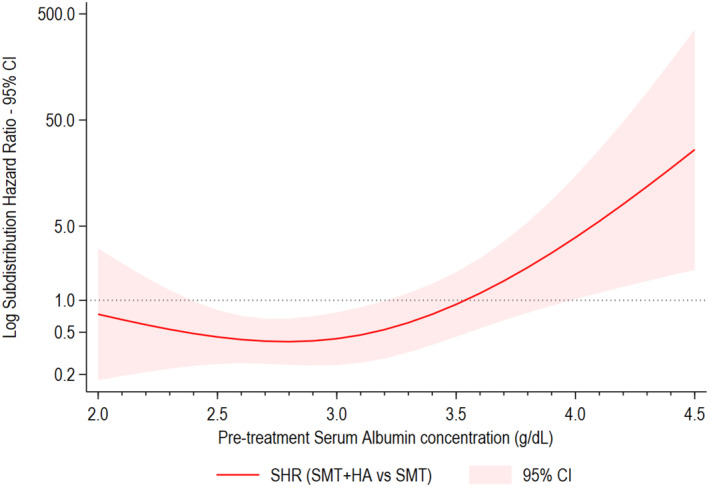
Treatment effect of baseline serum albumin. Estimated subdistribution hazard ratio (SHR) for death comparing SMT + HA versus SMT across baseline serum albumin values, derived from a multivariable Fine and Gray model treating liver transplantation as a competing event. Baseline albumin was modeled continuously using restricted cubic splines and the model was adjusted for MELD‐Na, Child–Pugh score, age, and viral etiology. The red line represents the estimated SHR and the shaded area the 95% confidence interval.

To formally evaluate treatment‐effect heterogeneity across the full spectrum of baseline SA, we used a multivariable Fine and Gray model in which baseline SA was modeled using restricted cubic splines. This model identified a significant non‐linear treatment‐by‐albumin interaction (global interaction test *p* = 0.010), indicating heterogeneity of treatment effect across baseline SA levels (Figure 1).

This finding was confirmed by internal validation using 1000 bootstrap resamples (global interaction test *p* = 0.046).

In the spline‐based Fine and Gray model, the estimated sHR for SMT + HA versus SMT indicated that the upper limit of the range of statistically demonstrable benefit occurred at approximately 3.2 g/dL (sHR 0.53, 95% CI 0.28–0.99). Accordingly, baseline SA ≤ 3.2 g/dL versus > 3.2 g/dL was used as an exploratory threshold for subsequent analyses.

**FIGURE 2:**
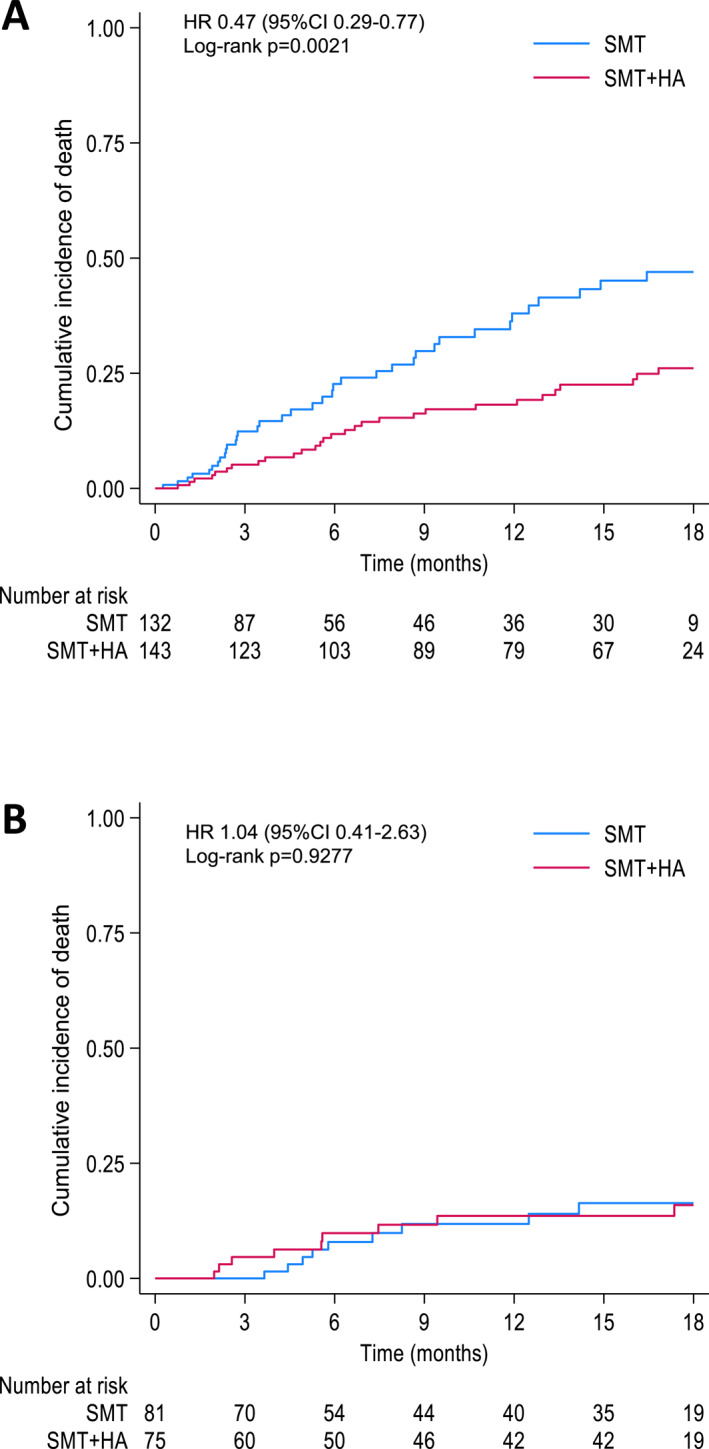
Kaplan‐Meier estimates of cumulative incidence of death in patients with baseline serum albumin level ≤ 3.2 g/dL (Panel A) or > 3.2 g/dL (Panel B) randomized in the Standard Medical Treatment (SMT) or SMT plus human albumin (SMT + HA) arms.

Applying this cut‐off, patients receiving LTA with baseline SA concentration ≤ 3.2 g/dL had a significantly greater 18‐month survival than those randomized to SMT alone (HR 0.47 [95% CI 0.29–0.77]; 74% [95% CI 64%–81%] vs. 53% [95% CI 40%–64%], *p* = 0.0021) (Figure 2A). Conversely, no significant survival benefit was demonstrated among patients with baseline serum albumin concentrations > 3.2 g/dL (HR 1.04, 95% CI 0.41–2.63) (Figure 2B), who had a low mortality rate in both treatment groups.

Given prior analyses of the ANSWER trial showing that on‐treatment SA levels at 1 month are significantly associated with survival [10], we examined whether SA concentrations reached after 1 month of treatment could discriminate the benefit from LTA. Interestingly, the treatment‐by‐albumin interaction was not statistically significant (global interaction test *p* = 0.859), suggesting that SA levels reached after 1 month of treatment do not discriminate the relative benefit of SMT + HA versus SMT.

Conversely, the 1‐month SA concentration remained strongly associated with subsequent mortality in models without interaction (global spline test *p* = 0.001), supporting its prognostic value.

### 3.2 Baseline Clinical Features in Patients With Baseline SA Concentration ≤ 3.2 g/dL or > 3.2 g/dL: Comparison Between SMT + HA versus SMT Alone

The baseline demographics, clinical characteristics, medical history and laboratory parameters of patients with baseline SA ≤ 3.2 g/dL or > 3.2 g/dL in the SMT or SMT + HA arms are reported in Table 2. Age, gender, etiology of cirrhosis, grade of ascites, hemodynamic parameters, clinical history, and prognostic scores were similar between the SMT + HA and SMT arms, either in patients with baseline SA concentration ≤ 3.2 g/dL or in those with baseline SA concentration > 3.2 g/dL. Among laboratory parameters, in patients with baseline SA concentration ≤ 3.2 g/dL, a significantly lower hemoglobin concentration was observed in the SMT group, while in patients with baseline SA concentration > 3.2 g/dL, a significantly higher leukocyte and platelet counts, and a lower INR were found in the SMT + HA group.

**TABLE 2:** Baseline characteristics of patients with baseline serum albumin level ≤ 3.2 g/dL and patients with baseline serum albumin level > 3.2 g/dL randomized in the standard medical treatment (SMT) or the SMT plus albumin (SMT + HA) arm.

	Baseline serum albumin level ≤ 3.2 g/dL			Baseline serum albumin level > 3.2 g/dL		
	SMT 132 (48%)	SMT + HA 143 (52%)	*p*‐value	SMT 81 (52%)	SMT + HA 75 (48%)	*p*‐value
Demographic data						
Age (years)	62 ± 11	61 ± 11	0.611	61 ± 12	61 ± 12	0.984
Male sex (*n*, %)	94 (71.2%)	91 (63.6%)	0.181	56 (69.1%)	55 (73.3%)	0.563
Aetiology of cirrhosis			0.328			0.255
Viral (*n*, %)	51 (38.6%)	51 (35.7%)		24 (29.6%)	21 (28.0%)	
Alcohol (*n*, %)	37 (28.0%)	38 (26.6%)		32 (39.5%)	25 (33.3%)	
MASH (*n*, %)	6 (4.5%)	3 (2.1%)		6 (7.4%)	5 (6.7%)	
Alcohol + viral (*n*, %)	19 (14.4%)	23 (16.1%)		4 (4.9%)	13 (17.3%)	
Alcohol + MASH (*n*, %)	7 (5.3%)	4 (2.8%)		4 (4.9%)	2 (2.7%)	
Other (*n*, %)	12 (9.1%)	24 (16.8%)		11 (13.6%)	9 (12.0%)	
Clinical features at inclusion						
Grade of ascites			0.424			0.529
Ascites grade 1 (*n*, %)	9 (6.8%)	6 (4.2%)		12 (14.8%)	8 (10.7%)	
Ascites grade 2 (*n*, %)	97 (73.5%)	114 (79.7%)		55 (67.9%)	57 (76.0%)	
Ascites grade 3 (*n*, %)	26 (19.7%)	23 (16.1%)		14 (17.3%)	10 (13.3%)	
Hyponatremia (sNa^+^ < 135 mmol/L)	59 (44.7%)	53 (37.1%)	0.198	15 (18.5%)	22 (29.3%)	0.113
Hepatic encephalopathy grade I/II	16 (12.1%)	16 (11.2%)	0.810	2 (2.5%)	5 (6.7%)	0.206
MAP (mmHg)	83 ± 9	84 ± 9	0.303	87 ± 10	86 ± 10	0.254
HR (bpm)	71 (62–78)	70 (64–78)	0.749	70 (64–80)	72 (66–80)	0.348
Medical history						
Oesophageal varices (*n*, %)	81 (61.4%)	97 (67.8%)	0.262	61 (75.3%)	47 (62.7%)	0.087
Prior overt HE (*n*, %)	43 (32.6%)	41 (28.7%)	0.482	14 (17.3%)	14 (18.7%)	0.822
Prior gastrointestinal bleeding (*n*, %)	21 (15.9%)	17 (11.9%)	0.334	14 (17.3%)	10 (13.3%)	0.494
Previous SBP (*n*, %)	10 (7.6%)	15 (10.5%)	0.401	9 (11.1%)	3 (4.0%)	0.096
Active list for LT (*n*, %)	11 (8.3%)	12 (8.4%)	0.986	6 (7.4%)	5 (6.7%)	0.857
Diuretics						
Antialdosteronic drugs (mg/day)	200 (200–300)	200 (200–300)	0.403	200 (200–200)	200 (200–300)	0.110
Furosemide (mg/day)	50 (25–75)	50 (25–75)	0.439	50 (25–50)	50 (25–50)	0.941
Laboratory data at inclusion						
Hb (g/dL)	11.2 ± 1.8	11.6 ± 1.8	0.033	12.0 ± 1.6	12.1 ± 1.9	0.900
WBC (10^9^/L)	4.79 (3.67–6.65)	5.10 (3.76–6.40)	0.502	4.71 (3.70–6.10)	5.27 (4.20–6.90)	0.042
Platelet count (10^9^/L)	89 (59–135)	86 (61–126)	0.901	90 (60–109)	113 (72–142)	0.003
Serum sodium (mmol/L)	135 (132–138)	135 (133–138)	0.315	137 (135–139)	137 (133–139)	0.932
Serum bilirubin (mg/dL)	2.13 (1.39–3.14)	1.98 (1.40–3.60)	0.808	1.59 (1.00–2.30)	1.33 (0.72–2.26)	0.116
Serum creatinine (mg/dL)	0.96 (0.80–1.16)	0.93 (0.80–1.10)	0.478	0.99 (0.80–1.15)	0.90 (0.77–1.14)	0.457
Serum albumin (g/dL)	2.80 (2.60–3.01)	2.80 (2.53–3.10)	0.890	3.50 (3.40–3.80)	3.70 (3.50–3.90)	0.098
INR	1.40 (1.22–1.63)	1.38 (1.24–1.54)	0.608	1.28 (1.16–1.42)	1.20 (1.14–1.33)	0.024
Prognostic scores						
Child–pugh score	8 (7–10)	9 (8–10)	0.558	7 (6–8)	7 (6–8)	0.249
MELD score	14 (11–17)	14 (11–17)	0.594	12 (9–14)	11 (9–13)	0.125
MELD‐Na score	18 (14–20)	16 (13–20)	0.134	14 (11–17)	14 (10–16)	0.362

*Note:* Data is reported by median and interquartile range or absolute frequency and percentage (%) as appropriate.

Abbreviations: Hb: hemoglobin; HR: heart rate; HE: hepatic encephalopathy; INR: International Normalized Ratio; LT: liver transplantation; MASH: Metabolic Associated steatohepatitis; MELD‐Na: Model for End‐stage Liver disease incorporating serum sodium; MAP: mean arterial pressure; MELD: Model for End‐stage Liver disease; SBP: spontaneous bacterial peritonitis; WBC: white blood cells.

### 3.3 Causes of Study Termination in Patients With Baseline SA Concentration ≤ 3.2 g/dL or > 3.2 g/dL: Comparison Between SMT + HA versus SMT

Regarding the prespecified cause of study termination, patients with baseline SA ≤ 3.2 g/dL randomized to receive SMT had a more frequent need for three or more therapeutic paracenteses per month and died more than those on SMT + HA, while no differences were observed for TIPS placement or LT (Supporting Information S1). In patients with baseline SA > 3.2 g/dL, the only significant difference was the higher number of patients needing three or more therapeutic paracenteses in the SMT arm as compared to the SMT + HA arm (Supporting Information S1).

### 3.4 Predictors of Mortality in Patients With Baseline SA Concentration ≤ 3.2 g/dL or > 3.2 g/dL: Comparison Between SMT + HA Versus SMT

**FIGURE 3:**
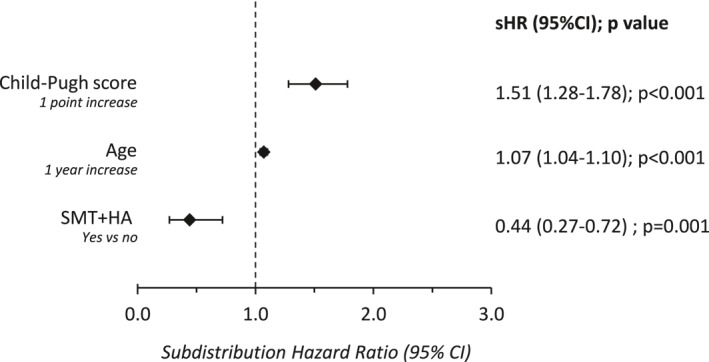
Multivariable Fine and Gray competing‐risk regression analysis, considering liver transplantation as a competing event, of factors associated with 18‐month all‐cause mortality in patients with baseline serum albumin ≤ 3.2 g/dL. Parameters included in the initial model were treatment arm, age, viral etiology, Child–Pugh score, and MELD‐Na score. Results are reported as subdistribution hazard ratios (sHRs) with 95% confidence intervals (CI).

In the group with baseline SA concentration ≤ 3.2 g/dL, 66 (24%) patients (37 from the SMT and 29 from the SMT + HA arm) died during the 18‐month follow‐up period. Parameters significantly associated with 18‐month mortality were receiving SMT alone, older age, viral etiology, Child Pugh, MELD and MELD‐Na scores, and serum sodium and creatinine (Supporting Information S1). Multivariable Fine and Gray competing risk regression analysis, considering LT as a competing event, showed that older age and higher Child–Pugh score were independently associated with an increased sHR for death during the 18‐month follow‐up, while HA treatment was the only factor independently associated with a significantly lower risk of death (Figure 3).

Among subjects with baseline SA concentration > 3.2 g/dL, only 18 (12%) patients died (9 from the SMT and 9 from the SMT + HA arm) during the 18‐month follow‐up. Contrary to what was observed in patients with baseline SA ≤ 3.2 g/dL, only the severity of cirrhosis, as reflected by prognostic scores and serum bilirubin level, was associated with mortality (Supporting Information S1).

### 3.5 Response of Ascites in Patients With Baseline SA Concentration ≤ 3.2 g/dL or > 3.2 g/dL: Comparison Between SMT + HA Versus SMT

**FIGURE 4:**
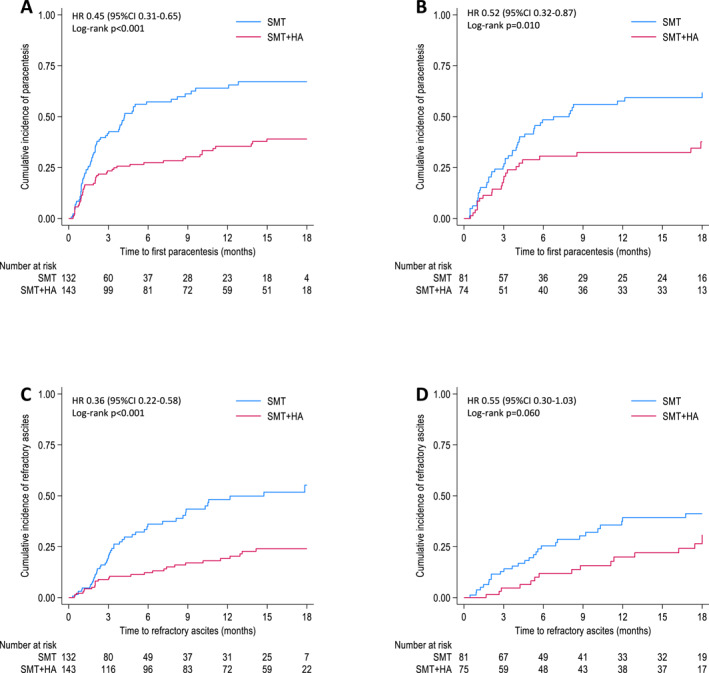
Kaplan‐Meier estimate of cumulative incidence of paracentesis in patients with baseline serum albumin level ≤ 3.2 g/dL (Panel A) or > 3.2 g/dL (Panel B) and cumulative incidence of refractory ascites in patients with baseline serum albumin level ≤ 3.2 g/dL (Panel C) or > 3.2 g/dL (Panel D) randomized in the Standard Medical Treatment (SMT) or SMT plus human albumin (SMT + HA) arms.

The number of patients requiring at least one LVP was significantly lower among those receiving SMT + HA than SMT alone, either with baseline SA concentration ≤ 3.2 g/dL (48 [34%] vs. 71 [54%], *p* = 0.001) or > 3.2 g/dL (23 [31%] vs. 45 [56%], *p* = 0.002). Similarly, the cumulative incidence of therapeutic paracenteses during the 18‐month follow‐up was significantly lower in the SMT + HA arm irrespective of baseline SA concentration (Figure 4, panels A and B).

Finally, patients with baseline SA concentration ≤ 3.2 g/dL randomized to SMT + HA developed refractory ascites defined according to the ICA criteria less frequently than those receiving SMT alone. A similar trend was observed in patients with baseline SA concentration > 3.2 g/dL, but the difference did not reach statistical significance (Figure 4, panels C and D).

### 3.6 Complications of Cirrhosis and Hospital Admissions With Baseline SA Concentration ≤ 3.2 g/dL or > 3.2 g/dL: Comparison Between SMT + HA Versus SMT

**FIGURE 5:**
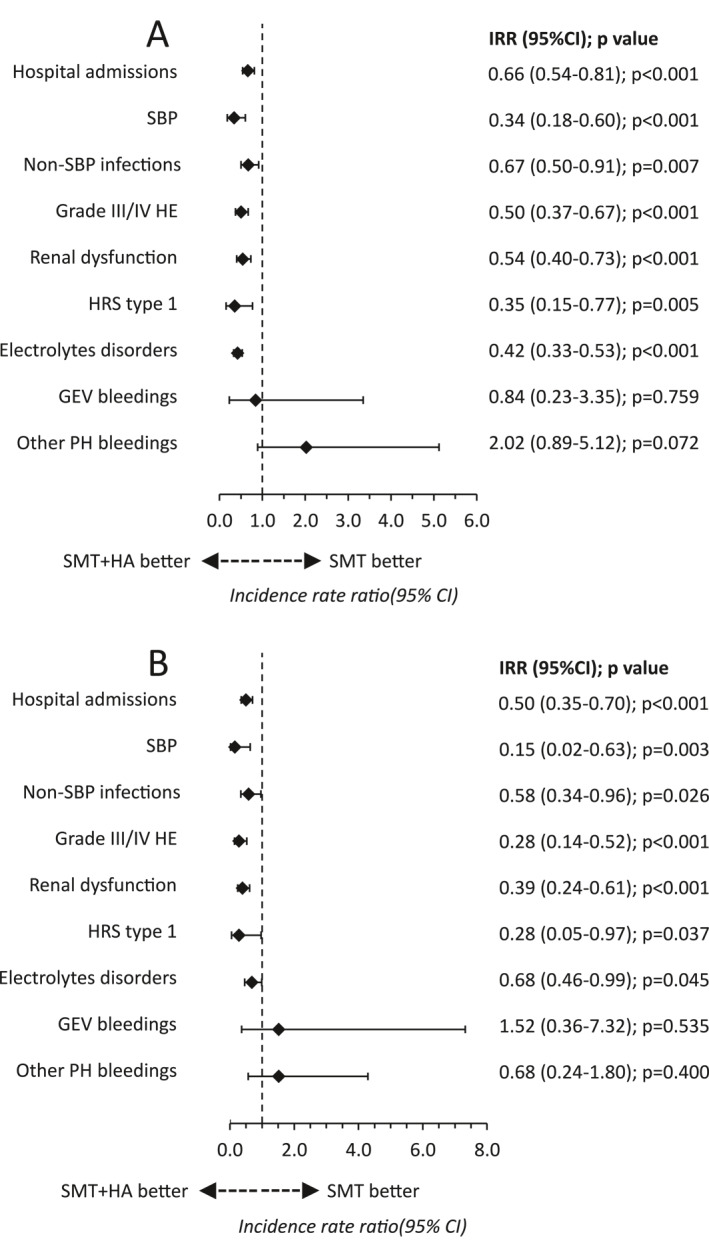
Incidence rate ratio (IRR) with 95% confidence interval of cirrhosis complications and hospital admissions in patients randomized in the standard medical treatment (SMT) or SMT plus human albumin (SMT + HA) arm with baseline serum albumin level ≤ 3.2 g/dL (Panel A) or > 3.2 g/dL (Panel B). GEV: gastroesophageal; HE: hepatic encephalopathy; HRS: hepatorenal syndrome; PH: portal hypertensive; SBP: spontaneous bacterial peritonitis.

Patients with baseline SA concentration ≤ 3.2 g/dL receiving SMT + HA had a lower incidence rate (IR) of complications of cirrhosis as compared to patients receiving SMT alone. Indeed, the Incidence Rate Ratio (IRR) was significantly in favor of the SMT + HA arm, except for gastroesophageal variceal bleeding or other portal hypertensive bleedings (Supporting Information S1 and Figure 5, panel A). Consistently, the IR of hospital admission in the SMT + HA arm was lower than that in the SMT arm, leading to an IRR significantly in favor of HA (Supporting Information S1 and Figure 5, panel A).

A similar lower IR of complications and hospital admissions in the SMT + HA was also observed in patients with baseline SA > 3.2 g/dL as compared to SMT alone. As a result, the IRR was significantly in favor of the SMT + HA arm for all variables, except for portal hypertensive‐related bleedings (Supporting Information S1 and Figure 5, panel B).

### 3.7 Comparison of Clinical Features and Outcomes Between Patients With Baseline SA Concentration ≤ 3.2 g/dL Versus > 3.2 g/dL

To understand why the better response of ascites and the lower incidence of complications was not associated with an improvement of survival in patients with a baseline SA concentration > 3.2 g/dL in patients receiving HA, we compared the baseline characteristics and clinical outcomes of patients presenting a baseline SA concentration ≤ 3.2 g/dL or > 3.2 g/dL in both SMT + HA and SMT arms.

While no differences were detected regarding age, sex and etiology of cirrhosis, as expected, patients with baseline SA concentration ≤ 3.2 g/dL were characterized by a more advanced disease as compared to those with a baseline SA concentration > 3.2 g/dL (Supporting Information S1).

Consistently, the secondary efficacy endpoints were significantly better in patients with higher baseline SA concentrations. Indeed, except for bleedings and renal impairment, the incidence rates of the other complications as well as the hospital admissions during the 18‐month follow‐up were significantly lower in patients with baseline SA concentration > 3.2 g/dL when compared to those with baseline SA concentration ≤ 3.2 g/dL (Supporting Information S1).

## 4 Discussion

This post hoc analysis of the ANSWER trial provides new insights into the debated role of LTA therapy in patients with decompensated cirrhosis and uncomplicated ascites. Although hypoalbuminemia is a well‐known independent negative prognostic factor [11, 12, 13], the observation that pre‐treatment SA concentration can be useful for predicting clinical benefit among patients receiving LTA is novel. This analysis demonstrates that a meaningful survival benefit of LTA emerges with at least mild‐to‐moderate hypoalbuminemia before treatment, but it could not be demonstrated in patients with normal or near‐normal SA concentrations. Conversely, improvements in ascites control and reductions in other complications occur independently of pre‐treatment SA levels.

It is important to underline that the lack of a survival benefit in patients with baseline SA > 3.2 g/dL should be interpreted with caution. In this subgroup, the remarkably low 18‐month mortality (12%) limits the statistical power to detect an improvement in survival without a significantly larger sample size. In this context, preliminary findings from the multicentre phase III PRECIOSA trial showed reduced rates of cirrhosis‐related complications with LTA (including SBP and HRS) and an early signal toward improved transplant‐free survival (3‐month HR 0.58). However, the primary endpoint of 1‐year transplant‐free survival was not achieved [14]. If meaningful survival benefit is largely limited to patients with baseline hypoalbuminemia, failure to stratify participants by baseline SA could dilute the overall effect on 1‐year survival while preserving improvements in morbidity.

Another novel finding of this study is that, in patients receiving LTA, SA functions as a biomarker for survival but with a different role depending on whether it is assessed before treatment or after 1 month of therapy. Baseline SA levels can identify patients with differing responses to LTA, whereas their prognostic value appears largely attenuated by treatment itself, as previously reported in the ANSWER trial [1]. In contrast, SA measured after 1 month retains a prognostic role, as no interaction with treatment effect can be demonstrated.

Taken together, these findings indicate that LTA therapy should be viewed primarily as a replacement strategy aimed at correcting a physiological deficit rather than targeting a specific molecular pathway. Baseline SA reflects the degree of deficiency at treatment initiation and therefore helps identify patients more likely to derive a survival benefit at least when a fixed‐dose of albumin is given, as it occurred in the ANSWER study. Consistent with this perspective, a practical goal would be to determine the SA concentration at which LTA should be initiated. In line with this reasoning, we identified in our cohort a threshold above which LTA does not appear to confer a survival benefit. However, we recognize that data‐driven cut‐off selection may be vulnerable to overfitting, underscoring the need for external validation in independent cohorts to ensure broader applicability. Therefore, rather than endorsing a single, definitive SA cut‐off, the main clinical implication of our findings is that LTA provides both survival and morbidity benefits in patients with at least mild‐to‐moderate hypoalbuminemia, whereas in those with normal or near‐normal serum albumin levels, its advantage appears to be largely confined to reducing morbidity. This distinction may help inform patient selection in settings where cost and logistical limitations constrain the prescription of LTA. Moreover, baseline SA level may be considered as a stratification factor in future randomized trials of long‐term albumin to account for potential heterogeneity of treatment effect observed in our analyses.

Consequently, if albumin is administered as replacement therapy, the primary objective is to restore physiological serum albumin levels [3, 10]. In our cohort, using the HA dosing regimen adopted in the ANSWER trial, approximately 75% of patients achieved and maintained SA concentrations above 3.5 g/dL, and 35% exceeded 4.0 g/dL, with a median on‐treatment value of around 3.8–3.9 g/dL [10]. It may therefore be hypothesized that an appropriate therapeutic target for LTA is to attain a normal SA concentration, typically defined as > 3.5 g/dL according to internationally accepted laboratory reference ranges (3.5–5.0 g/dL). Nonetheless, given that several studies suggest that on‐treatment SA levels approaching 4.0 g/dL are associated with more favourable clinical and pathophysiological outcomes [4, 10], pursuing higher therapeutic targets may be sometimes appropriate, taking into account the patient's response to LTA, the availability of alternative treatment options, and relevant cost or logistical considerations. However, the negative results of the ATTIRE trial also indicate that the underlying clinical condition and treatment duration are critical factors, since targeting a pre‐specified serum albumin level for up to 2 weeks in patients with acute decompensation did not translate into similar clinical benefits [15]. Therefore, based on currently available data, the rationale of using albumin to restore physiological levels should be restricted to the context of LTA administration.

This study has several limitations. First, this post hoc analysis was not pre‐specified and may be underpowered for certain subgroup and secondary endpoint analyses. Specifically, the subgroup with baseline SA > 3.2 g/dL included relatively few patients and was characterized by a low mortality rate (12%), which limits the statistical power to definitively exclude a survival benefit in this specific population. Second, this analysis shares the limitations of the ANSWER trial itself, including its open‐label design, while retaining the strengths of a large, pragmatic, multicentre, randomized clinical trial enrolling more than 400 patients [1]. Another relevant consideration is the evolving epidemiology of chronic liver disease. The decline in hepatitis C‐related cirrhosis in Western countries, alongside the rising prevalence of metabolic dysfunction‐associated steatotic liver disease (MASLD) and alcohol‐related liver disease, may affect the generalizability of our findings to current clinical practice. However, the results were not influenced by the effects of viral cures because the study period preceded the widespread availability of direct‐acting antivirals.

In conclusion, this post hoc analysis of the ANSWER database indicates that pre‐treatment SA concentration can predict differential outcomes in patients with decompensated cirrhosis and uncomplicated ascites receiving LTA, thereby helping clinicians identify the subgroup most likely to derive maximal benefit from this therapy.

## Author Contributions

Conception or design of the work (E.P., G.Z., M.B., P.C.), Data collection (all the authors), Data analysis and interpretation (E.P., M.B.), Drafting the article (E.P., G.Z., M.B., P.C.), Critical revision of the article (E.P., G.Z., C.A., M.B., M.B., P.C.). All authors have approved the submitted manuscript.

## Funding

The ANSWER trial was funded by the competitive Grant FARM6P824B from the Italian Medicine Agency (AIFA).

## Conflicts of Interest

The following authors disclose conflicts of interest: GZ: Grifols SA, Octapharma SA (speaking bureau). SP: Plasma Protein Therapeutics Association, Boehringer Ingelheim, Resolution therapeutics (Consultant); Grifols SA and MEDSCAPE (Sponsored lectures). PA: Biovie (advisory board and patent), CSL Behring (speaker invitation and travel grant), Grifols (speaker invitation), Kedrion (speaker invitation), Biomarin (advisory board), GenFit SA (advisory board). MBe: Grifols SA (speaking bureau and advisory board), CSL Behring GmbH (speaking bureau). PC: Grifols SA (speaking bureau and advisory boards), Octapharma SA (speaking bureau ), CSL Behring (speaking bureau), Takeda (speaking bureau); Abbvie (speaking bureau and advisory boards). All the other authors disclose no conflicts of interest.

## Supporting information


Supporting Information S1

## Data Availability

The data that support the findings of this study are available on request from the corresponding author. The data are not publicly available due to privacy or ethical restrictions.
