# Correction to “Pre‐treatment Serum Albumin Concentration Predicts Clinical Benefit of Long‐Term Albumin in Patients With Cirrhosis and Ascites”

**DOI:** 10.1002/ueg2.70280

**Published:** 2026-08-19

Pompili E, Zaccherini G, Nardelli S, Piano S, Alessandria C, Neri S, Foschi FG, Levantesi F, Airoldi A, Simone L, Svegliati‐Baroni G, Fagiuoli S, Marra F, Cozzolongo R, Iannone G, Biselli M, Riggio O, Angeli P, Bernardi M, Baldassarre M, Caraceni P; ANSWER Study Investigators. Pre‐Treatment Serum Albumin Concentration Predicts Clinical Benefit of Long‐Term Albumin in Patients With Cirrhosis and Ascites. *United European Gastroenterology Journal*. July 2026; 14(6):e70258. https://doi.org/10.1002/ueg2.70258. PMID: 42435328; PMCID: PMC13355858.

In the published version of this article, the institutional affiliation of Dr. Raffaele Cozzolongo was published incompletely. The affiliation did not include the designation “IRCCS”, which is part of the official institutional name.

The affiliation should read:

National Institute of Gastroenterology IRCCS “Saverio de Bellis”, Castellana Grotte, Bari, Italy.

This correction concerns only the institutional affiliation of the author. The scientific content of the article remains unchanged.

We apologize for this error.

