# Supplementary material for: Pre‐Treatment Serum Albumin Concentration Predicts Clinical Benefit of Long‐Term Albumin in Patients With Cirrhosis and Ascites
Source: United European Gastroenterol J. 2026 Jul 11;14(6):e70258. doi: 10.1002/ueg2.70258 (PMC13355858; doi:10.1002/ueg2.70258)
Supplement: Supplementary file 1 — Supporting Information S1 [file UEG2-14-e70258-s001.docx]

# **SUPPLEMENTARY DATA**

**PRE-TREATMENT SERUM ALBUMIN CONCENTRATION PREDICTS CLINICAL BENEFIT**

**OF LONG-TERM ALBUMIN IN PATIENTS WITH CIRRHOSIS AND ASCITES**

## **Complete list of the ANSWER study Investigators**

Marco Domenicali, Manuel Tufoni, Ferdinando A Giannone, Agnese Antognoli, Greta Tedesco, Francesco Palmese (Department of Medical and Surgical Sciences, University of Bologna); Manuela Merli, Chiara Pasquale, Stefania Gioia (Department of Translational and Precision Medicine, Sapienza University of Rome); Silvano Fasolato, Antonietta Sticca, Marta Cavallin (Unit of Internal Medicine and Hepatology, Department of Medicine, University of Padua); Daniela Campion, Alessandro Risso, Alida Andrealli, Giorgio M Saracco, Chiara Elia (Division of Gastroenterology and Hepatology, Città della Salute e della Scienza Hospital, University of Turin); Loredana Prestianni, Federica Fidone, Daniela Maiorca, Agostino Rizzotto (Department of Clinical and Experimental Medicine, University of Catania); Federica Mirici Cappa, Arianna Lanzi, Elga Neri, Anna Visani (Internal Medicine, Hospital of Faenza, AUSL of Romagna); Antonio Mastroianni (Internal Medicine, Hospital of Bentivoglio, AUSL of Bologna); Aldo Airoldi, Giovanni Perricone, Alberto B Alberti, Lucia Cesarini, Chiara Mazzarelli, Marcello Vangeli, Raffaella Viganò (Liver Unit, Department of Hepatology and Gastroenterology, Niguarda Hospital, Milan); Marco Marzioni, Francesca Capretti, Alba Kostandini (Department of Gastroenterology, Polytechnic University of Marche, Ancona); Giulia Magini, Maria Colpani (Gastroenterology and Transplant Hepatology, Papa Giovanni XXIII Hospital, Bergamo); Giacomo Laffi, Tommaso Gabbani, Maria Marsico, Roberto G Romanelli (Department of Experimental and Clinical Medicine, University of Florence); Marianna Zappimbulso, Josè Petruzzi (Division of Gastroenterology, National Institute of Gastroenterology S De Bellis, Castellana Grotte); Vito Di Marco, Vincenza Calvaruso (Unit of Gastroenterology and Hepatology, Biomedical Department of Internal and Specialistic Medicine, University of Palermo); Vincenzo Sangiovanni, Giovanni Parrella (AORN dei Colli “Cotugno” Hospital of Naples); Nicola Caporaso, Filomena Morisco, Francesco Auriemma, Maria Guarino (Department of Clinical Medicine and Surgery, Gastroenterology Unit, Federico II University of Naples); Pierluigi Toniutto, Fabio Pugliese (Internal Medicine, Department of Medical Area, University of Udine); Antonio Gasbarrini, Annalisa Tortora (Gastroenterology, Gemelli Foundation, Cattolica University, Rome); Pietro Leo, Rosanna De Marco (Gastroenterology Unit, Hospital of Cosenza); Mario Angelico, Francesco De Leonardis, Alessandra Pecchioli, Piera Rossi (Liver Unit, Tor Vergata University, Rome); Giovanni Raimondo, Irene Cacciola (Division of Clinical and Molecular Hepatology, University Hospital of Messina); Gianfranco Elia, Elisa Negri (Infectious Diseases and Hepatology, University Hospital of Parma, Italy); Marcello Dallio, Carmelina Loguercio, Alessandro Federico (University of Campania Luigi Vanvitelli, Naples); Dario Conte, Sara Massironi (Gastroenterology and Endoscopy Unit, Foundation IRCSS Ca’ Granda Ospedale Maggiore Policlinico, Milan); Giorgio Ballardini, Natascia Celli (AUSL of Romagna, Internal Medicine, Hospital of Rimini); Maria Rendina, Roberto Bringiotti, Nicola Maurizio Castellaneta (Section of Gastroenterology, Department of Emergency and Organ Transplantation, University of Bari); Francesco Salerno (Internal Medicine, IRCSS Policlinico San Donato, University of Milan); Sergio Boccia (Gastroenterology Unit, University Hospital, Ferrara); Riccardo Guarisco (Internal Medicine, S. Sebastiano General Hospital, Frascati); Alessandra Galioto (Internal Medicine, Hospital of Dolo, Azienda Unità Locale Socio-Sanitaria Serenissima, Mestre).

## **Supplementary table 1**

Factors associated with 18-month mortality in patients with baseline serum albumin (SA) concentration ≤3.2g/dL or >3.2g/dL in the whole population (n = 431).

|  | **Baseline SA ≤3.2 g/dL**  *N=275* | | **Baseline SA >3.2 g/dL**  *N=156* | |
| --- | --- | --- | --- | --- |
|  | *sHR (95%CI)* | *P value* | *sHR (95%CI)* | *P value* |
| Long-term albumin treatment | 0.49 (0.30 - 0.80) | 0.005 | 1.05 (0.41 - 2.65) | 0.914 |
| Female sex | 1.44 (0.87 - 2.38) | 0.146 | 0.86 (0.30 - 2.43) | 0.787 |
| Age | 1.05 (1.02 - 1.07) | <0.001 | 1.01 (0.97 - 1.05) | 0.585 |
| Viral etiology | 1.82 (1.12 - 2.94) | 0.014 | 0.86 (0.30 - 2.38) | 0.773 |
| Alcohol etiology | 0.62 (0.33-1.17) | 0.141 | 1.10 (0.43 - 2.82) | 0.840 |
| MASH etiology | 1.74 (0.55 - 5.51) | 0.342 | 1.67 (0.39 - 7.13) | 0.486 |
| Viral and alcohol etiology | 0.71 (0.34 - 1.47) | 0.364 | 1.32 (0.29 - 5.94) | 0.714 |
| Viral and MASH etiology | 0.99 (0.33 - 2.92) | 0.991 | - | . |
| Other etiology | 0.68 (0.31 - 1.47) | 0.332 | 0.91 (0.21 - 3.93) | 0.900 |
| MAP | 0.98 (0.95 - 1.01) | 0.313 | 1.00 (0.96 - 1.05) | 0.683 |
| Heart rate | 0.99 (0.97 - 1.01) | 0.633 | 1.01 (0.97 - 1.06) | 0.438 |
| Child Pugh score | 1.36 (1.16 - 1.59) | <0.001 | 1.59 (1.32 - 1.91) | <0.001 |
| MELD score | 1.09 (1.03 - 1.15) | 0.002 | 1.17 (1.08 - 1.25) | <0.001 |
| MELD-Na score | 1.10 (1.04 - 1.16) | 0.001 | 1.11 (1.03 - 1.19) | 0.003 |
| Leucocyte | 1.00 (0.91 - 1.10) | 0.867 | 0.99 (0.82 - 1.21) | 0.987 |
| Hemoglobin | 0.88 (0.77 - 1.01) | 0.072 | 0.89 (0.67 - 1.19) | 0.465 |
| Platelets | 0.99 (0.99 – 1.00) | 0.808 | 1.00 (0.99 – 1.00) | 0.899 |
| Albumin | 0.50 (0.24 - 1.02) | 0.059 | 0.28 (0.01 - 4.28) | 0.365 |
| Creatinine | 2.84 (1.02 - 7.87) | 0.044 | 1.86 (0.21 - 16.46) | 0.576 |
| Sodium | 0.92 (0.86 - 0.98) | 0.012 | 0.99 (0.89 - 1.09) | 0.847 |
| Potassium | 1.13 (0.73 - 1.74) | 0.569 | 0.74 (0.28 - 1.94) | 0.552 |
| Bilirubin | 1.06 (0.98 - 1.15) | 0.104 | 1.28 (1.13 - 1.46) | 0.001 |
| INR | 2.10 (0.92 - 4.79) | 0.076 | 2.83 (0.85 - 9.44) | 0.090 |
| Previous paracentesis | 1.26 (0.77 - 2.05) | 0.350 | 1.38 (0.53 - 3.58) | 0.496 |
| Hyponatremia | 1.37 (0.84 - 2.22) | 0.200 | 1.46 (0.52 - 4.09) | 0.464 |

*Abbreviations: MASH: Metabolic Associated steatohepatitis; INR: International Normalized Ratio; MAP: mean arterial pressure; MELD: Model for End-stage Liver disease; MELD-Na: Model for End-stage Liver disease incorporating serum sodium.*

## **Supplementary table 2**

Incidence rate (IR) with 95% confidence interval of cirrhosis complications and hospital admissions in patients randomized in the standard medical treatment (SMT) or SMT plus human albumin (SMT+HA) arm with baseline serum albumin level ≤3.2 g/dL or >3.2 g/dL.

|  |  | ***SMT IR (95%CI)*** | ***SMT+HA IR (95%CI)*** |
| --- | --- | --- | --- |
| **SBP** | **≤3.2 g/dL** | 0.41 (0.28-0.57) | 0.14 (0.08-0.21) |
|  | **>3.2 g/dL** | 0.19 (0.10-0.32) | 0.03 (0.01-0.10) |
| **Non SBP** infection | **≤3.2 g/dL** | 1.06 (0.85-1.31) | 0.72 (0.58-0.87) |
|  | **>3.2 g/dL** | 0.59 (0.43-0.79) | 0.34 (0.22-0.50) |
| **Grade III/IV HE** | **≤3.2 g/dL** | 1.20 (0.97-1.45) | 0.60 (0.47-0.74) |
|  | **>3.2 g/dL** | 0.64 (0.48-0.85) | 0.18 (0.09-0.30) |
| **Renal dysfunction** | **≤3.2 g/dL** | 1.14 (0.92-1.39) | 0.61 (0.49-0.75) |
|  | **>3.2 g/dL** | 0.95 (0.74-1.20) | 0.37 (0.24-0.53) |
| **HRS type 1** | **≤3.2 g/dL** | 0.23 (0.14-0.36) | 0.08 (0.04-0.14) |
|  | **>3.2 g/dL** | 0.15 (0.07-0.26) | 0.04 (0.01-0.12) |
| **Electrolyte disorders** | **≤3.2 g/dL** | 2.16 (1.86-2.50) | 0.91 (0.76-1.08) |
|  | **>3.2 g/dL** | 0.90 (0.70-1.14) | 0.61 (0.45-0.82) |
| **GEV bleedings** | **≤3.2 g/dL** | 0.06 (0.02-0.14) | 0.05 (0.02-0.10) |
|  | **>3.2 g/dL** | 0.05 (0.02-0.14) | 0.08 (0.03-0.18) |
| **Other PH bleedings** | **≤3.2 g/dL** | 0.10 (0.04-0.19) | 0.19 (0.13-0.28) |
|  | **>3.2 g/dL** | 0.11 (0.05-0.21) | 0.16 (0.08-0.29) |
| **Hospital admissions** | **≤3.2 g/dL** | 2.20 (1.89-2.54) | 1.45 (1.26-1.67) |
|  | **>3.2 g/dL** | 1.39 (1.14-1.69) | 0.69 (0.52-0.91) |

*Abbreviations: SBP: spontaneous bacterial peritonitis; HE: hepatic encephalopathy; HRS: hepatorenal syndrome; GEV: gastroesophageal; PH: portal hypertensive.*

## **Supplementary table 3**

*Baseline clinical features of patients randomized to the standard medical treatment (SMT) or SMT plus albumin (SMT+HA) arms compared according to their baseline serum albumin concentration level: ≤3.2 g/dL or >3.2 g/dL.*

|  | | **SMT** | | | **SMT+HA** | | |
| --- | --- | --- | --- | --- | --- | --- | --- |
|  | | **SA ≤3.2g/dL**  *n = 132 (62%)* | **SA >3.2g/dL**  *N = 81 (38%)* | P-value | **SA ≤3.2g/dL**  *143 (66%)* | **SA >3.2g/dL**  *75 (34%)* | P-value |
| **Demographic data** | |  |  |  |  |  |  |
|  | Age (years) | 62 ± 11 | 61 ± 12 | 0.909 | 61 ± 11 | 61 ± 12 | 0.742 |
|  | Male sex (n, %) | 94 (71.2%) | 56 (69.1%) | 0.747 | 91 (63.6%) | 55 (73.3%) | 0.148 |
| **Aetiology of cirrhosis** | |  |  | 0.108 |  |  | 0.387 |
|  | Viral (n, %) | 51 (38.6%) | 24 (29.6%) |  | 51 (35.7%) | 21 (28.0%) |  |
|  | Alcohol (n, %) | 37 (28.0%) | 32 (39.5%) |  | 38 (26.6%) | 25 (33.3%) |  |
|  | MASH (n, %) | 6 (4.5%) | 6 (7.4%) |  | 3 (2.1%) | 5 (6.7%) |  |
|  | Alcohol + viral (n, %) | 19 (14.4%) | 4 (4.9%) |  | 23 (16.1%) | 13 (17.3%) |  |
|  | Alcohol + NASH (n, %) | 7 (5.3%) | 4 (4.9%) |  | 4 (2.8%) | 2 (2.7%) |  |
|  | Other (n, %) | 12 (9.1%) | 11 (13.6%) |  | 24 (16.8%) | 9 (12.0%) |  |
| **Clinical features at inclusion** | |  |  |  |  |  |  |
|  | Grade of ascites |  |  | 0.163 |  |  | 0.170 |
|  | - Ascites grade 1 (n, %) | 9 (6.8%) | 12 (14.8%) |  | 6 (4.2%) | 8 (10.7%) |  |
|  | - Ascites grade 2 (n, %) | 97 (73.5%) | 55 (67.9%) |  | 114 (79.7%) | 57 (76.0%) |  |
|  | - Ascites grade 3 (n, %) | 26 (19.7%) | 14 (17.3%) |  | 23 (16.1%) | 10 (13.3%) |  |
|  | Hyponatremia (Na^+^ <135 mmol/l) | 59 (44.7%) | 15 (18.5%) | <0.001 | 53 (37.1%) | 22 (29.3%) | 0.254 |
|  | Hepatic encephalopathy grade I/II | 16 (12.1%) | 2 (2.5%) | 0.014 | 16 (11.2%) | 5 (6.7%) | 0.282 |
|  | MAP (mmHg) | 83 ± 9 | 87 ± 10 | 0.002 | 84 ± 9 | 86 ± 9 | 0.357 |
|  | HR (bpm) | 71 (62-78) | 70 (64-80) | 0.966 | 70 (64-78) | 72 (66-80) | 0.235 |
| **Medical History** | |  |  |  |  |  |  |
|  | Presence of oesophageal varices (n, %) | 81 (61.4%) | 61 (75.3%) | 0.036 | 97 (67.8%) | 47 (62.7%) | 0.444 |
|  | Previous overt HE (n, %) | 43 (32.6%) | 14 (17.3%) | 0.014 | 41 (28.7%) | 14 (18.7%) | 0.106 |
|  | Previous gastrointestinal bleeding (n, %) | 21 (15.9%) | 14 (17.3%) | 0.793 | 17 (11.9%) | 10 (13.3%) | 0.758 |
|  | Previous SBP (n, %) | 10 (7.6%) | 9 (11.1%) | 0.380 | 15 (10.5%) | 3 (4.0%) | 0.098 |
|  | Active list for LT (n, %) | 11 (8.3%) | 6 (7.4%) | 0.809 | 12 (8.4%) | 5 (6.7%) | 0.652 |
| **Diuretics** | |  |  |  |  |  |  |
|  | Antialdosteronic drugs (mg/day) | 200 (200-300) | 200 (200-200) | 0.442 | 200 (200-300) | 200 (200-300) | 0.834 |
|  | Furosemide (mg/day) | 50 (25-75) | 50 (25-50) | 0.108 | 50 (25-75) | 50 (25-50) | 0.265 |
| **Laboratory data at inclusion** | |  |  |  |  |  |  |
|  | Hb (g/dL) | 11.2 ± 1.8 | 12.0 ± 1.6 | <0.001 | 11.6 ± 1.8 | 12.1 ± 1.9 | 0.113 |
|  | WBC (10^9^/L) | 4.79 (3.67-6.65) | 4.71 (3.70-6.10) | 0.665 | 5.10 (3.76-6.40) | 5.27 (4.20-6.90) | 0.228 |
|  | Platelet count (10^9^/L) | 89 (59-136) | 90 (60-109) | 0.701 | 86 (61-126) | 113 (72-142) | 0.008 |
|  | Serum sodium (mmol/L) | 135 ± 4 | 137 ± 3 | 0.004 | 135 ± 4 | 136 ± 5 | 0.102 |
|  | Serum bilirubin (mg/dL) | 2.13 (1.39-3.14) | 1.59 (1.00-2.30) | 0.002 | 1.98 (1.40-3.60) | 1.33 (0.72-2.26) | <0.001 |
|  | Serum creatinine (mg/dL) | 0.96 (0.80-1.16) | 0.99 (0.80-1.15) | 0.912 | 0.93 (0.80-1.10) | 0.90 (0.77-1.14) | 0.735 |
|  | Serum albumin (g/dL) | 2.79 ± 0.30 | 3.61 ± 0.25 | <0.001 | 2.78 ± 0.34 | 3.70 ± 0.32 | <0.001 |
|  | INR | 1.40 (1.22-1.63) | 1.28 (1.16-1.42) | <0.001 | 1.38 (1.24-1.54) | 1.20 (1.14-1.33) | <0.001 |
| **Prognostic scores** | |  |  |  |  |  |  |
|  | Child-Pugh score | 8 (7-10) | 7 (6-8) | <0.001 | 9 (8-10) | 7 (6-8) | <0.001 |
|  | MELD score | 14 (11-17) | 12 (9-14) | <0.001 | 14 (11-17) | 11 (9-13) | <0.001 |
|  | MELD-Na score | 18 (14-20) | 14 (11-17) | <0.001 | 16 (13-20) | 14 (10-16) | <0.001 |

*Data is reported by median and interquartile range or absolute frequency and percentage (%) as appropriate.* *Abbreviations: MASH: Metabolic Associated steatohepatitis; HE: hepatic encephalopathy; SBP: spontaneous bacterial peritonitis; LT: liver transplantation; Hb: haemoglobin; WBC: white blood cells; INR: International Normalized Ratio; MAP: mean arterial pressure; HR: heart rate; MELD: Model for End-stage Liver disease; MELD-Na: Model for End-stage Liver disease incorporating serum sodium.*

## **Supplementary figure 1**


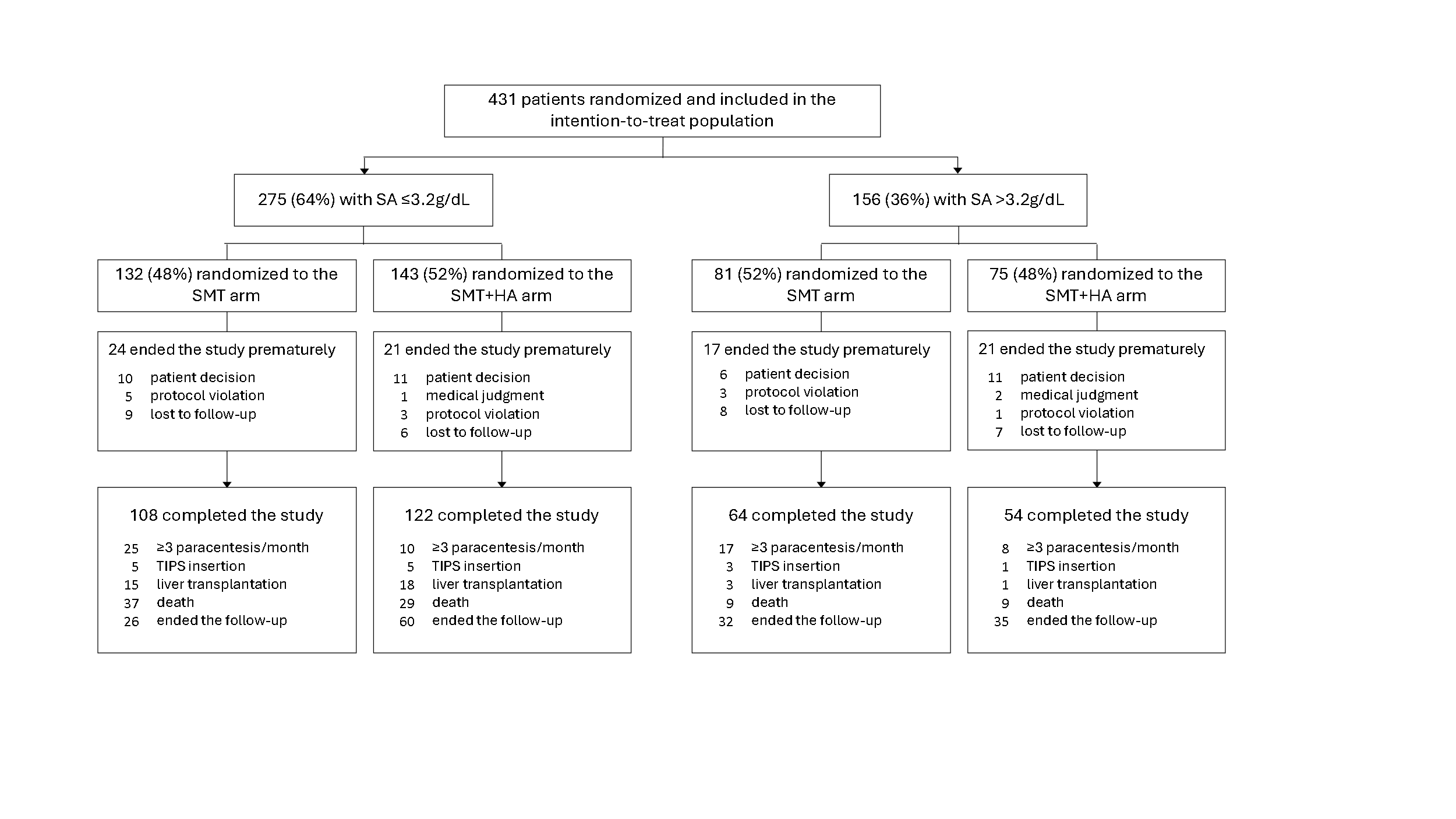


**Supplementary figure 1.** *Reasons for early study termination and prespecified causes of study termination in patients with baseline serum albumin level ≤3.2 g/dL or >3.2 g/dL randomized in the Standard Medical Treatment (SMT) or SMT plus human albumin (SMT+HA) arms.*

## **Supplementary figure 2**

**
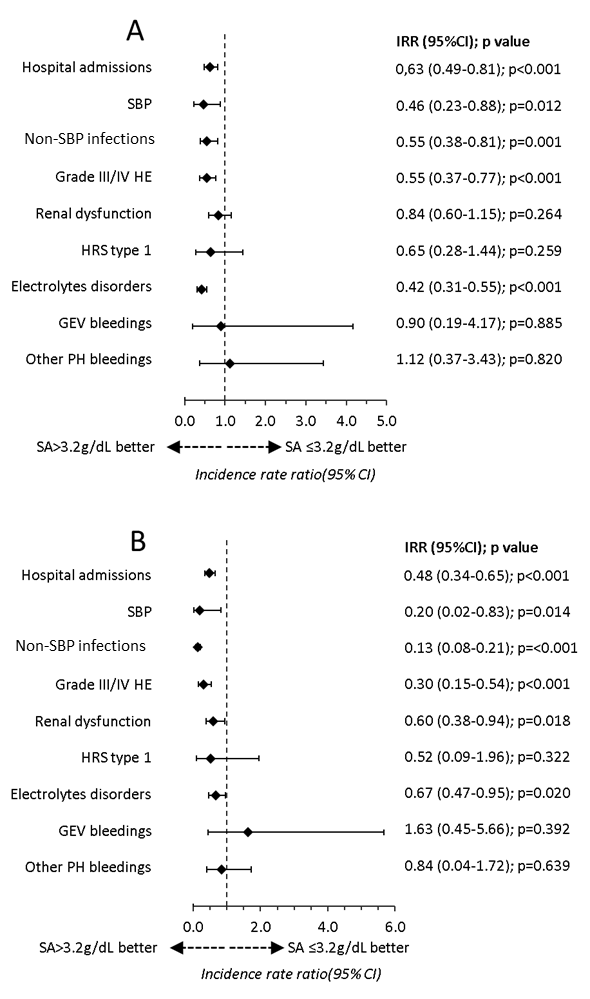
**

**Supplementary figure 2.** *Incidence rate ratio (IRR) with 95% confidence interval of cirrhosis complications and hospital admissions in patients randomized in the standard medical treatment (SMT) (Panel A) or SMT plus human albumin (SMT+HA) (Panel B) arm with baseline serum albumin level ≤3.2 g/dL or >3.2 g/dL. Abbreviations: SBP: spontaneous bacterial peritonitis; HE: hepatic encephalopathy; HRS: hepatorenal syndrome; GEV: gastroesophageal; PH: portal hypertensive.*
